# 1-Chloro­acetyl-3-isopropyl-*r*-2,*c*-6-diphenyl­piperidin-4-one

**DOI:** 10.1107/S160053680905497X

**Published:** 2010-01-09

**Authors:** K. Ravichandran, P. Ramesh, P. Jeganathan, S. Ponnuswamy, M. N. Ponnuswamy

**Affiliations:** a Centre of Advanced Study in Crystallography and Biophysics, University of Madras, Guindy Campus, Chennai 600 025, India; b Department of Chemistry, Government Arts College (Autonomous), Coimbatore 641 018, India

## Abstract

In the title compound, C_22_H_24_ClNO_2_, the piperidine ring adopts a distorted boat conformation. The dihedral angle between the two phenyl rings is 83.2 (1)°. In the crystal, the mol­ecules are linked into chains running along the *b* axis by C—H⋯O hydrogen bonds. The Cl atom of the chloro­acetyl group is disordered over two positions with occupancies of 0.66 (2) and 0.34 (2).

## Related literature

For general background to piperidine derivatives, see: El-Subbagh *et al.* (2000 ▶); Jerom & Spencer (1988 ▶); Perumal *et al.* (2001 ▶); Hagenbach & Gysin (1952 ▶); Mobio *et al.* (1989 ▶); Katritzky & Fan (1990 ▶); Ganellin & Spickett (1965 ▶). For asymmetry and puckering parameters, see: Nardelli (1983 ▶); Cremer & Pople (1975 ▶). For hydrogen-bond motifs, see: Bernstein *et al.* (1995 ▶). For the synthesis, see: Venkatraj *et al.* (2008 ▶).
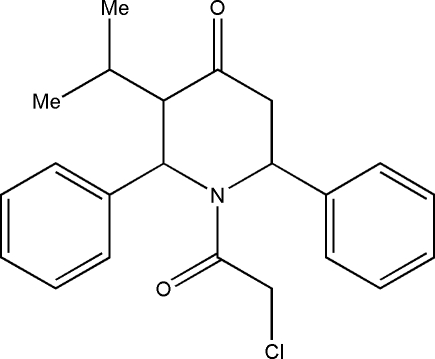


## Experimental

### 

#### Crystal data

C_22_H_24_ClNO_2_
*M* *_r_* = 369.87
Monoclinic, 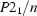

*a* = 10.3415 (12) Å
*b* = 9.0243 (9) Å
*c* = 21.438 (2) Å
β = 90.894 (3)°
*V* = 2000.5 (4) Å^3^
*Z* = 4
Mo *K*α radiation
μ = 0.21 mm^−1^
*T* = 293 K
0.23 × 0.23 × 0.20 mm

#### Data collection

Bruker SMART APEXII area-detector diffractometer
Absorption correction: multi-scan (*SADABS*; Bruker, 2008 ▶) *T*_min_ = 0.954, *T*_max_ = 0.960
19037 measured reflections
4965 independent reflections
3634 reflections with *I* > 2σ(*I*)
*R*_int_ = 0.026

#### Refinement

*R*[*F* ^2^ > 2σ(*F* ^2^)] = 0.049
*wR*(*F* ^2^) = 0.152
*S* = 1.05
4965 reflections
247 parameters
H-atom parameters constrained
Δρ_max_ = 0.51 e Å^−3^
Δρ_min_ = −0.24 e Å^−3^

### 

Data collection: *APEX2* (Bruker, 2008 ▶); cell refinement: *SAINT* (Bruker, 2008 ▶); data reduction: *SAINT*; program(s) used to solve structure: *SHELXS97* (Sheldrick, 2008 ▶); program(s) used to refine structure: *SHELXL97* (Sheldrick, 2008 ▶); molecular graphics: *ORTEP-3* (Farrugia, 1997 ▶); software used to prepare material for publication: *SHELXL97* and *PLATON* (Spek, 2009 ▶).

## Supplementary Material

Crystal structure: contains datablocks global, I. DOI: 10.1107/S160053680905497X/ci2993sup1.cif

Structure factors: contains datablocks I. DOI: 10.1107/S160053680905497X/ci2993Isup2.hkl

Additional supplementary materials:  crystallographic information; 3D view; checkCIF report

## Figures and Tables

**Table 1:** Hydrogen-bond geometry (Å, °)

*D*—H⋯*A*	*D*—H	H⋯*A*	*D*⋯*A*	*D*—H⋯*A*
C6—H6⋯O1^i^	0.98	2.57	3.504 (2)	160
C8—H8*C*⋯O1^i^	0.96	2.25	3.203 (2)	174

Symmetry code: (i) 
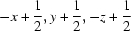
.

**Fig. 1.**
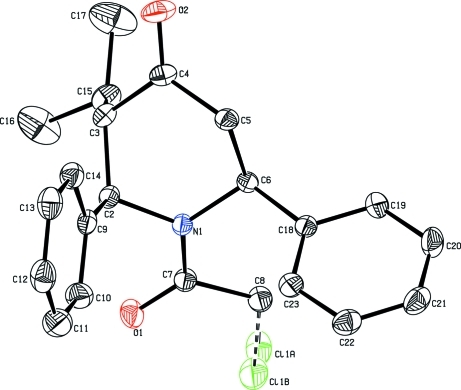
The molecular structure of the title compound. Displacement ellipsoids are drawn at the 30% probability level. H atoms have been omitted for clarity. Both disorder components are shown.

**Fig. 2.**
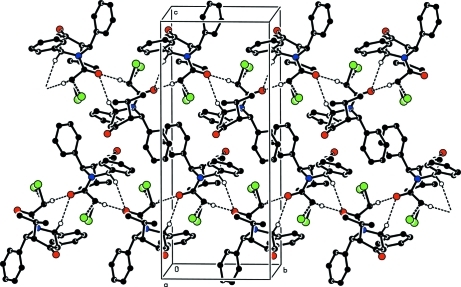
The crystal packing of the title compound viewed along the a axis. H atoms not involved in hydrogen bonding have been omitted for clarity.

## supplementary crystallographic information

### Comment

Piperidine derivatives gained considerable importance owing to their varied biological properties such as antiviral, antitumour (El-Subbagh *et al.*, 2000), analgesic (Jerom & Spencer, 1988), local anaesthetic (Perumal *et al.*, 2001; Hagenbach & Gysin, 1952), antimicrobial, bactericidal, fungicidal, herbicidal, insecticidal, antihistaminic, anti-inflammatory, anticancer, *CNS* stimulant and depressant activities (Mobio *et al.*, 1989; Katritzky & Fan, 1990; Ganellin & Spickett, 1965). In view of these importance and to ascertain the molecular conformation, crystallographic study of the title compound has been carried out.

The *ORTEP* plot of the title molecule is shown in Fig.1. The piperidine ring adopts a distorted boat conformation with the puckering parameters (Cremer & Pople, 1975) and asymmetry parameters (Nardelli, 1983) of q_2_ = 0.661 (2) Å, q_3_ = -0.057 (2) Å, φ_2_ = 257.1 (1)° and Δ_s_(C2 and C5)= 20.2 (2)°. The sum of the bond angles around the atom N1 (358.8°) of the piperidine ring is in accordance with the *sp*^2^ hybridization.

The crystal packing is stabilized by C—H···O intermolecular interactions which link the molecules into chains running along the *b* axis. These hydrogen bonds form R^1^_2_(7) ring motifs (Bernstein *et al.*, 1995).

### Experimental

To a solution of r-2,c-6-diphenyl-3-isopropylpiperidin-4-one (2.93 g) in anhydrous benzene (60 ml) was added triethylamine (5.57 ml) and chloroacetylchloride (3.18 ml). The reaction mixture was allowed to stirr at room temperature for 2 h. The resulting solution was washed with sodium bicarbonate solution (10%) and water. Then the organic layer was dried over anhydrous sodium sulfate, evaporated and crystallized from benzene-petroleum ether (60–80°C) in the ratio of 9:1 (Venkatraj *et al.*, 2008).

### Refinement

The Cl atom of the chloroacetyl group is disordered over two positions with refined occupancies of 0.662 (18) and 0.338 (18). H atoms were positioned geometrically (C-H = 0.93–0.98 Å) and allowed to ride on their parent atoms, with U_iso_(H) = 1.5*U*_eq_(C_methyl_) and 1.2*U*_eq_(C).

### Crystal data

**Table:**

C_22_H_24_ClNO_2_	*F*(000) = 784
*M**_r_* = 369.87	*D*_x_ = 1.228 Mg m^−^^3^
Monoclinic, *P*2_1_/*n*	Mo *K*α radiation, λ = 0.71073 Å
Hall symbol: -P 2yn	Cell parameters from 2052 reflections
*a* = 10.3415 (12) Å	θ = 1.9–28.3°
*b* = 9.0243 (9) Å	µ = 0.21 mm^−^^1^
*c* = 21.438 (2) Å	*T* = 293 K
β = 90.894 (3)°	Block, colourless
*V* = 2000.5 (4) Å^3^	0.23 × 0.23 × 0.20 mm
*Z* = 4	

### Data collection

**Table:**

Bruker SMART APEXII area-detector diffractometer	4965 independent reflections
Radiation source: fine-focus sealed tube	3634 reflections with *I* > 2σ(*I*)
graphite	*R*_int_ = 0.026
ω and φ scans	θ_max_ = 28.3°, θ_min_ = 1.9°
Absorption correction: multi-scan (*SADABS*; Bruker, 2008)	*h* = −13→13
*T*_min_ = 0.954, *T*_max_ = 0.960	*k* = −11→12
19037 measured reflections	*l* = −28→26

### Refinement

**Table:**

Refinement on *F*^2^	Primary atom site location: structure-invariant direct methods
Least-squares matrix: full	Secondary atom site location: difference Fourier map
*R*[*F*^2^ > 2σ(*F*^2^)] = 0.049	Hydrogen site location: inferred from neighbouring sites
*wR*(*F*^2^) = 0.152	H-atom parameters constrained
*S* = 1.05	*w* = 1/[σ^2^(*F*_o_^2^) + (0.073*P*)^2^ + 0.4534*P*] where *P* = (*F*_o_^2^ + 2*F*_c_^2^)/3
4965 reflections	(Δ/σ)_max_ = 0.001
247 parameters	Δρ_max_ = 0.51 e Å^−^^3^
0 restraints	Δρ_min_ = −0.24 e Å^−^^3^

### Special details

**Table:**

Geometry. All e.s.d.'s (except the e.s.d. in the dihedral angle between two l.s. planes) are estimated using the full covariance matrix. The cell e.s.d.'s are taken into account individually in the estimation of e.s.d.'s in distances, angles and torsion angles; correlations between e.s.d.'s in cell parameters are only used when they are defined by crystal symmetry. An approximate (isotropic) treatment of cell e.s.d.'s is used for estimating e.s.d.'s involving l.s. planes.
Refinement. Refinement of *F*^2^ against ALL reflections. The weighted *R*-factor *wR* and goodness of fit *S* are based on *F*^2^, conventional *R*-factors *R* are based on *F*, with *F* set to zero for negative *F*^2^. The threshold expression of *F*^2^ > σ(*F*^2^) is used only for calculating *R*-factors(gt) *etc*. and is not relevant to the choice of reflections for refinement. *R*-factors based on *F*^2^ are statistically about twice as large as those based on *F*, and *R*- factors based on ALL data will be even larger.

### Fractional atomic coordinates and isotropic or equivalent isotropic displacement parameters (Å^2^)

**Table:**

	*x*	*y*	*z*	*U*_iso_*/*U*_eq_	Occ. (<1)
Cl1A	0.4290 (5)	0.2714 (5)	0.19072 (9)	0.0742 (9)	0.662 (18)
Cl1B	0.4685 (12)	0.2335 (12)	0.2045 (7)	0.097 (2)	0.338 (18)
O1	0.28951 (14)	0.09562 (14)	0.28351 (6)	0.0649 (4)	
O2	−0.07198 (13)	0.48645 (19)	0.43316 (7)	0.0758 (5)	
N1	0.23607 (12)	0.28293 (14)	0.34870 (6)	0.0385 (3)	
C2	0.13248 (14)	0.19074 (18)	0.37710 (7)	0.0416 (3)	
H2	0.1191	0.1067	0.3488	0.050*	
C3	0.00443 (15)	0.2766 (2)	0.37661 (7)	0.0460 (4)	
H3	−0.0585	0.2166	0.3992	0.055*	
C4	0.01886 (16)	0.4218 (2)	0.41130 (7)	0.0494 (4)	
C5	0.15466 (15)	0.47860 (19)	0.41923 (7)	0.0446 (4)	
H5A	0.1910	0.4381	0.4576	0.053*	
H5B	0.1513	0.5854	0.4240	0.053*	
C6	0.24626 (14)	0.44202 (17)	0.36585 (7)	0.0382 (3)	
H6	0.2197	0.5009	0.3294	0.046*	
C7	0.30043 (16)	0.22482 (18)	0.29965 (7)	0.0444 (4)	
C8	0.39016 (18)	0.3296 (2)	0.26547 (8)	0.0524 (4)	
H8A	0.4694	0.3406	0.2898	0.063*	0.662 (18)
H8B	0.3495	0.4263	0.2627	0.063*	0.662 (18)
H8C	0.3412	0.4109	0.2485	0.063*	0.338 (18)
H8D	0.4536	0.3690	0.2941	0.063*	0.338 (18)
C9	0.17615 (15)	0.12393 (18)	0.43941 (7)	0.0447 (4)	
C10	0.2790 (2)	0.0264 (2)	0.43950 (10)	0.0638 (5)	
H10	0.3203	0.0062	0.4022	0.077*	
C11	0.3221 (2)	−0.0420 (3)	0.49375 (12)	0.0769 (6)	
H11	0.3916	−0.1073	0.4927	0.092*	
C12	0.2619 (2)	−0.0130 (3)	0.54915 (10)	0.0709 (6)	
H12	0.2906	−0.0582	0.5858	0.085*	
C13	0.1599 (2)	0.0821 (2)	0.55003 (9)	0.0656 (5)	
H13	0.1190	0.1015	0.5875	0.079*	
C14	0.11594 (18)	0.1510 (2)	0.49564 (8)	0.0541 (4)	
H14	0.0459	0.2154	0.4970	0.065*	
C15	−0.05025 (19)	0.2994 (3)	0.30908 (9)	0.0619 (5)	
H15	0.0186	0.3409	0.2835	0.074*	
C16	−0.0902 (4)	0.1544 (3)	0.28166 (14)	0.1154 (12)	
H16A	−0.0205	0.0847	0.2860	0.173*	
H16B	−0.1114	0.1673	0.2382	0.173*	
H16C	−0.1645	0.1177	0.3031	0.173*	
C17	−0.1643 (3)	0.4015 (4)	0.30539 (14)	0.1232 (13)	
H17A	−0.1407	0.4963	0.3223	0.185*	
H17B	−0.2340	0.3606	0.3289	0.185*	
H17C	−0.1912	0.4129	0.2626	0.185*	
C18	0.38149 (15)	0.49029 (18)	0.38534 (7)	0.0409 (3)	
C19	0.41717 (19)	0.6350 (2)	0.37469 (9)	0.0560 (4)	
H19	0.3597	0.6991	0.3546	0.067*	
C20	0.5382 (2)	0.6861 (2)	0.39369 (10)	0.0674 (5)	
H20	0.5611	0.7842	0.3866	0.081*	
C21	0.62369 (19)	0.5928 (3)	0.42275 (10)	0.0656 (5)	
H21	0.7050	0.6270	0.4351	0.079*	
C22	0.58976 (18)	0.4492 (3)	0.43370 (9)	0.0631 (5)	
H22	0.6483	0.3856	0.4533	0.076*	
C23	0.46858 (17)	0.3976 (2)	0.41585 (8)	0.0511 (4)	
H23	0.4455	0.3002	0.4244	0.061*	

### Atomic displacement parameters (Å^2^)

**Table:**

	*U*^11^	*U*^22^	*U*^33^	*U*^12^	*U*^13^	*U*^23^
Cl1A	0.1037 (17)	0.0687 (12)	0.0512 (8)	−0.0259 (10)	0.0369 (7)	−0.0192 (6)
Cl1B	0.104 (4)	0.077 (3)	0.112 (4)	−0.017 (3)	0.069 (3)	−0.030 (3)
O1	0.0795 (9)	0.0486 (7)	0.0674 (8)	−0.0137 (7)	0.0272 (7)	−0.0207 (6)
O2	0.0458 (7)	0.0976 (12)	0.0841 (10)	0.0165 (7)	0.0073 (7)	−0.0291 (9)
N1	0.0384 (6)	0.0373 (7)	0.0400 (6)	−0.0036 (5)	0.0062 (5)	−0.0048 (5)
C2	0.0383 (7)	0.0427 (8)	0.0440 (7)	−0.0064 (6)	0.0049 (6)	−0.0035 (6)
C3	0.0362 (8)	0.0569 (10)	0.0450 (8)	−0.0049 (7)	0.0034 (6)	−0.0001 (7)
C4	0.0422 (8)	0.0618 (11)	0.0444 (8)	0.0076 (8)	0.0042 (6)	−0.0040 (7)
C5	0.0448 (8)	0.0450 (9)	0.0441 (8)	0.0029 (7)	0.0059 (6)	−0.0079 (7)
C6	0.0395 (7)	0.0365 (8)	0.0388 (7)	0.0011 (6)	0.0025 (5)	−0.0034 (6)
C7	0.0463 (8)	0.0448 (9)	0.0424 (7)	−0.0033 (7)	0.0075 (6)	−0.0065 (6)
C8	0.0616 (10)	0.0507 (10)	0.0456 (8)	−0.0037 (8)	0.0173 (7)	−0.0050 (7)
C9	0.0424 (8)	0.0418 (9)	0.0500 (8)	−0.0109 (7)	0.0046 (6)	0.0026 (7)
C10	0.0636 (12)	0.0599 (12)	0.0682 (12)	0.0076 (10)	0.0117 (9)	0.0112 (9)
C11	0.0686 (13)	0.0721 (15)	0.0902 (16)	0.0093 (11)	0.0031 (11)	0.0279 (12)
C12	0.0746 (14)	0.0682 (13)	0.0694 (12)	−0.0138 (11)	−0.0121 (10)	0.0241 (10)
C13	0.0776 (14)	0.0677 (13)	0.0516 (10)	−0.0166 (11)	0.0048 (9)	0.0084 (9)
C14	0.0557 (10)	0.0535 (10)	0.0531 (9)	−0.0055 (8)	0.0062 (7)	0.0021 (8)
C15	0.0524 (10)	0.0820 (14)	0.0510 (9)	−0.0004 (10)	−0.0046 (8)	0.0034 (9)
C16	0.156 (3)	0.101 (2)	0.0880 (17)	0.026 (2)	−0.0594 (19)	−0.0347 (16)
C17	0.136 (3)	0.129 (3)	0.103 (2)	0.055 (2)	−0.057 (2)	−0.0268 (19)
C18	0.0414 (8)	0.0422 (8)	0.0393 (7)	−0.0018 (7)	0.0064 (6)	−0.0080 (6)
C19	0.0563 (10)	0.0439 (10)	0.0676 (11)	−0.0045 (8)	0.0000 (8)	−0.0062 (8)
C20	0.0657 (12)	0.0539 (11)	0.0829 (14)	−0.0205 (10)	0.0043 (10)	−0.0130 (10)
C21	0.0461 (10)	0.0804 (15)	0.0703 (12)	−0.0131 (10)	−0.0001 (8)	−0.0182 (11)
C22	0.0493 (10)	0.0743 (14)	0.0653 (11)	0.0028 (10)	−0.0079 (8)	−0.0049 (10)
C23	0.0477 (9)	0.0512 (10)	0.0542 (9)	0.0002 (8)	−0.0012 (7)	−0.0005 (7)

### Geometric parameters (Å, °)

**Table:**

Cl1A—C8	1.739 (3)	C10—H10	0.93
Cl1B—C8	1.774 (5)	C11—C12	1.375 (3)
O1—C7	1.221 (2)	C11—H11	0.93
O2—C4	1.207 (2)	C12—C13	1.360 (3)
N1—C7	1.3585 (19)	C12—H12	0.93
N1—C6	1.4853 (19)	C13—C14	1.391 (3)
N1—C2	1.4936 (19)	C13—H13	0.93
C2—C9	1.527 (2)	C14—H14	0.93
C2—C3	1.534 (2)	C15—C16	1.491 (3)
C2—H2	0.98	C15—C17	1.497 (3)
C3—C4	1.513 (2)	C15—H15	0.98
C3—C15	1.560 (2)	C16—H16A	0.96
C3—H3	0.98	C16—H16B	0.96
C4—O2	1.207 (2)	C16—H16C	0.96
C4—C5	1.502 (2)	C17—H17A	0.96
C5—C6	1.533 (2)	C17—H17B	0.96
C5—H5A	0.97	C17—H17C	0.96
C5—H5B	0.97	C18—C19	1.377 (2)
C6—C18	1.517 (2)	C18—C23	1.386 (2)
C6—H6	0.98	C19—C20	1.389 (3)
C7—C8	1.521 (2)	C19—H19	0.93
C8—H8A	0.97	C20—C21	1.364 (3)
C8—H8B	0.97	C20—H20	0.93
C8—H8C	0.96	C21—C22	1.363 (3)
C8—H8D	0.96	C21—H21	0.93
C9—C10	1.381 (3)	C22—C23	1.385 (3)
C9—C14	1.387 (2)	C22—H22	0.93
C10—C11	1.384 (3)	C23—H23	0.93
			
C7—N1—C6	122.07 (13)	C14—C9—C2	124.06 (16)
C7—N1—C2	117.63 (13)	C9—C10—C11	121.5 (2)
C6—N1—C2	119.08 (12)	C9—C10—H10	119.2
N1—C2—C9	111.86 (12)	C11—C10—H10	119.2
N1—C2—C3	109.90 (13)	C12—C11—C10	119.8 (2)
C9—C2—C3	116.66 (13)	C12—C11—H11	120.1
N1—C2—H2	105.9	C10—C11—H11	120.1
C9—C2—H2	105.9	C13—C12—C11	119.58 (19)
C3—C2—H2	105.9	C13—C12—H12	120.2
C4—C3—C2	110.82 (13)	C11—C12—H12	120.2
C4—C3—C15	111.93 (15)	C12—C13—C14	120.90 (19)
C2—C3—C15	111.93 (14)	C12—C13—H13	119.5
C4—C3—H3	107.3	C14—C13—H13	119.5
C2—C3—H3	107.3	C9—C14—C13	120.30 (19)
C15—C3—H3	107.3	C9—C14—H14	119.8
O2—C4—C5	121.54 (17)	C13—C14—H14	119.8
O2—C4—C3	122.57 (16)	C16—C15—C17	107.8 (2)
C5—C4—C3	115.86 (14)	C16—C15—C3	110.14 (19)
C4—C5—C6	115.44 (13)	C17—C15—C3	113.83 (18)
C4—C5—H5A	108.4	C16—C15—H15	108.3
C6—C5—H5A	108.4	C17—C15—H15	108.3
C4—C5—H5B	108.4	C3—C15—H15	108.3
C6—C5—H5B	108.4	C15—C16—H16A	109.5
H5A—C5—H5B	107.5	C15—C16—H16B	109.5
N1—C6—C18	114.04 (12)	H16A—C16—H16B	109.5
N1—C6—C5	110.53 (12)	C15—C16—H16C	109.5
C18—C6—C5	108.07 (12)	H16A—C16—H16C	109.5
N1—C6—H6	108.0	H16B—C16—H16C	109.5
C18—C6—H6	108.0	C15—C17—H17A	109.5
C5—C6—H6	108.0	C15—C17—H17B	109.5
O1—C7—N1	122.93 (15)	H17A—C17—H17B	109.5
O1—C7—C8	120.78 (14)	C15—C17—H17C	109.5
N1—C7—C8	116.28 (14)	H17A—C17—H17C	109.5
C7—C8—Cl1A	114.03 (15)	H17B—C17—H17C	109.5
C7—C8—Cl1B	109.9 (3)	C19—C18—C23	118.48 (16)
C7—C8—H8A	108.7	C19—C18—C6	118.35 (15)
Cl1A—C8—H8A	108.7	C23—C18—C6	123.11 (15)
Cl1B—C8—H8A	93.1	C18—C19—C20	120.57 (19)
C7—C8—H8B	108.7	C18—C19—H19	119.7
Cl1A—C8—H8B	108.7	C20—C19—H19	119.7
Cl1B—C8—H8B	126.7	C21—C20—C19	120.3 (2)
H8A—C8—H8B	107.6	C21—C20—H20	119.9
C7—C8—H8C	109.6	C19—C20—H20	119.9
Cl1A—C8—H8C	90.5	C22—C21—C20	119.88 (18)
Cl1B—C8—H8C	109.7	C22—C21—H21	120.1
H8A—C8—H8C	124.2	C20—C21—H21	120.1
C7—C8—H8D	109.7	C21—C22—C23	120.41 (19)
Cl1A—C8—H8D	122.4	C21—C22—H22	119.8
Cl1B—C8—H8D	109.7	C23—C22—H22	119.8
H8B—C8—H8D	89.9	C22—C23—C18	120.39 (18)
H8C—C8—H8D	108.2	C22—C23—H23	119.8
C10—C9—C14	117.86 (16)	C18—C23—H23	119.8
C10—C9—C2	118.04 (15)		
			
C7—N1—C2—C9	104.81 (16)	N1—C2—C9—C10	−62.6 (2)
C6—N1—C2—C9	−87.54 (16)	C3—C2—C9—C10	169.66 (16)
C7—N1—C2—C3	−123.94 (15)	N1—C2—C9—C14	119.83 (17)
C6—N1—C2—C3	43.72 (17)	C3—C2—C9—C14	−7.9 (2)
N1—C2—C3—C4	−57.76 (16)	C14—C9—C10—C11	−0.5 (3)
C9—C2—C3—C4	70.91 (18)	C2—C9—C10—C11	−178.19 (19)
N1—C2—C3—C15	67.97 (17)	C9—C10—C11—C12	0.0 (3)
C9—C2—C3—C15	−163.36 (14)	C10—C11—C12—C13	0.3 (3)
C2—C3—C4—O2	−157.42 (17)	C11—C12—C13—C14	−0.2 (3)
C15—C3—C4—O2	76.9 (2)	C10—C9—C14—C13	0.6 (3)
C2—C3—C4—C5	20.3 (2)	C2—C9—C14—C13	178.19 (16)
C15—C3—C4—C5	−105.41 (17)	C12—C13—C14—C9	−0.3 (3)
O2—C4—C5—C6	−149.50 (17)	C4—C3—C15—C16	−167.5 (2)
C3—C4—C5—C6	32.7 (2)	C2—C3—C15—C16	67.4 (2)
C7—N1—C6—C18	−62.87 (18)	C4—C3—C15—C17	−46.3 (3)
C2—N1—C6—C18	130.04 (13)	C2—C3—C15—C17	−171.4 (2)
C7—N1—C6—C5	175.14 (14)	N1—C6—C18—C19	150.61 (14)
C2—N1—C6—C5	8.06 (17)	C5—C6—C18—C19	−86.06 (17)
C4—C5—C6—N1	−47.51 (19)	N1—C6—C18—C23	−32.4 (2)
C4—C5—C6—C18	−172.94 (14)	C5—C6—C18—C23	90.93 (18)
C6—N1—C7—O1	−177.26 (16)	C23—C18—C19—C20	0.6 (3)
C2—N1—C7—O1	−10.0 (2)	C6—C18—C19—C20	177.69 (17)
C6—N1—C7—C8	3.6 (2)	C18—C19—C20—C21	0.5 (3)
C2—N1—C7—C8	170.82 (14)	C19—C20—C21—C22	−0.6 (3)
O1—C7—C8—Cl1A	20.2 (3)	C20—C21—C22—C23	−0.4 (3)
N1—C7—C8—Cl1A	−160.6 (3)	C21—C22—C23—C18	1.5 (3)
O1—C7—C8—Cl1B	−0.7 (7)	C19—C18—C23—C22	−1.6 (3)
N1—C7—C8—Cl1B	178.5 (7)	C6—C18—C23—C22	−178.55 (16)

### Hydrogen-bond geometry (Å, °)

**Table:**

*D*—H···*A*	*D*—H	H···*A*	*D*···*A*	*D*—H···*A*
C6—H6···O1^i^	0.98	2.57	3.504 (2)	160
C8—H8C···O1^i^	0.96	2.25	3.203 (2)	174

Symmetry codes: (i) −*x*+1/2, *y*+1/2, −*z*+1/2.
